# Opposing Effects of Chelidonine on Tyrosine and Serine Phosphorylation of STAT3 in Human Uveal Melanoma Cells

**DOI:** 10.3390/ijms222312974

**Published:** 2021-11-30

**Authors:** István Csomós, Péter Nagy, Csenge Filep, István Rebenku, Enikő Nizsalóczki, Tamás Kovács, György Vámosi, László Mátyus, Andrea Bodnár

**Affiliations:** Department of Biophysics and Cell Biology, Doctoral School of Molecular Medicine, Faculty of Medicine, Research Center for Molecular Medicine, University of Debrecen, Egyetem tér 1., H-4032 Debrecen, Hungary; csomos.istvan@med.unideb.hu (I.C.); nagyp@med.unideb.hu (P.N.); filep.csenge@med.unideb.hu (C.F.); rebenku.istvan@med.unideb.hu (I.R.); nizsaloe@med.unideb.hu (E.N.); kovacs.tamas@med.unideb.hu (T.K.); vamosig@med.unideb.hu (G.V.); lmatyus@med.unideb.hu (L.M.)

**Keywords:** STAT3 signaling, chelidonine, interleukin-6, flow cytometry, confocal microscopy, uveal melanoma

## Abstract

STAT3 is a transcription factor that regulates various cellular processes with oncogenic potential, thereby promoting tumorigenesis when activated uncontrolled. STAT3 activation is mediated by its tyrosine phosphorylation, triggering dimerization and nuclear translocation. STAT3 also contains a serine phosphorylation site, with a postulated regulatory role in STAT3 activation and G2/M transition. Interleukin-6, a major activator of STAT3, is present in elevated concentrations in uveal melanomas, suggesting contribution of dysregulated STAT3 activation to their pathogenesis. Here, we studied the impact of chelidonine on STAT3 signaling in human uveal melanoma cells. Chelidonine, an alkaloid isolated from *Chelidonium majus*, disrupts microtubules, causes mitotic arrest and provokes cell death in numerous tumor cells. According to our flow cytometry and confocal microscopy data, chelidonine abrogated IL-6-induced activation and nuclear translocation, but amplified constitutive serine phosphorylation of STAT3. Both effects were restricted to a fraction of cells only, in an all-or-none fashion. A partial overlap could be observed between the affected subpopulations; however, no direct connection could be proven. This study is the first proof on a cell-by-cell basis for the opposing effects of a microtubule-targeting agent on the two types of STAT3 phosphorylation.

## 1. Introduction

Signal transducer and activator of transcription-3 (STAT3) belongs to the family of latent transcription factors that reside predominantly in the cytoplasm of resting cells. Activation of STAT3 requires phosphorylation on the tyrosine 705 residue, which is followed by its dimerization and nuclear translocation. STAT3 in the nucleus initiates the transcription of a vast range of genes playing a pivotal role in various biological processes with oncogenic potential (e.g., cell proliferation, anti-apoptotic processes, cell migration, etc.). Consequently, uncontrolled (prolonged or constitutive) activation of STAT3 may lead to dysregulation of target gene expression, thereby promoting malignant transformation and tumor cell survival [1,2,3].

STAT3 is activated by multiple cytokines and growth factors, including the pro-inflammatory interleukin-6 (IL-6). IL-6 acts through a multimeric receptor complex comprising the cytokine-specific α-chain (IL-6Rα) and the signaling glycoprotein 130 (gp130) subunit shared with other members of the IL-6R family [4]. Binding of IL-6 to IL-6Rα triggers homo-dimerization and subsequent trans-phosphorylation of gp130 by Janus kinase (Jak)-family tyrosine kinases associated constitutively with gp130. Consequently, STAT3 is recruited to the receptor complex via its SH2 domain and becomes phosphorylated by the same tyrosine kinases. Under physiological conditions, tyrosine phosphorylation of STAT3 is controlled strictly by a couple of negative regulators (e.g., PIAS, SOCS and PTP) and has a transient time-course [5]. Dysregulation of the IL-6/gp130/STAT3 pathway is closely associated with the development of a variety of human malignancies, including melanomas [2,6,7,8].

Uveal melanoma is the most common primary intraocular malignancy in adults with a high susceptibility for metastasis resulting in poor prognosis [9,10]. Vitreous fluid of eyes with melanoma was shown previously to contain an elevated level of IL-6 (along with other cytokines/chemokines) compared to the healthy counterparts, suggesting that—via the prolonged activation of STAT3—it may contribute to the formation and maintenance of these tumors [11,12,13].

STAT3 can also be phosphorylated at the serine 727 residue, which is the target of various Ser/Thr kinases (e.g., MAP kinases, mTOR, CDK1, etc.) [14,15,16]. The exact role of serine phosphorylation in STAT3 function is still debated. Compelling evidence supports that it is required for the maximal transcriptional activity of STAT3 through recruiting transcriptional cofactors [16]. On the other hand, suppression/negative modulation of STAT3 activation by serine phosphorylation was reported in a variety of cell types, including human liver cancer cells [17]. The amount of pSer-STAT3 is elevated during mitosis, and so presumably has a crucial role in the onset of M phase [14].

Chelidonine, isolated from *Chelidonium majus* L., belongs to the family of benzophenatridine alkaloids. These compounds exhibit a wide range of pharmacological activities, including antitumor, anti-inflammatory and antimicrobial effects, among others [18,19]. Chelidonine was reported previously to exert cell growth inhibitory effects via the induction of apoptosis (and necrosis) in numerous human cancer cell types, including uveal melanomas [20,21,22,23]. Possibly acting at the colchicine-binding site of microtubules, chelidonine was shown to disrupt the structure and dynamics of the microtubular system and arrest cells in the G2/M phase of the cell cycle [22,24,25,26].

Microtubule-targeting agents (MTAs) constitute one of the major groups of chemotherapeutic drugs, widely used in combinational chemotherapy of solid tumors and hematological malignancies [27]. The common feature of their action is interfering with microtubular dynamics by either inhibiting or promoting polymerization of microtubules, which consequently leads to mitotic block and finally cell death. Accumulating evidence suggests that the actions of MTAs are much more complex than thought originally and not restricted to dividing cells [28]. Among others, several members of the MTA family were shown previously to interfere with STAT3 signaling, affecting both activation and serine phosphorylation of the transcription factor [29,30].

Taking into account the similarities between the actions of MTAs and chelidonine, the question arises whether chelidonine has an impact on STAT3 signaling. Therefore, we studied the potential effects of this alkaloid on serine and IL-6-induced tyrosine phosphorylation of STAT3 in human uveal melanoma cells using flow cytometry and confocal microscopy. These approaches make it possible to investigate intact or quasi-intact cells on a cell-by-cell basis, allowing us to reveal possible heterogeneities within the cell population, as well as to correlate the observed effects with each other.

According to our results, chelidonine increased the efficiency of constitutive serine phosphorylation, whereas it abrogated IL-6-induced tyrosine phosphorylation and nuclear translocation of STAT3 in the investigated human uveal melanoma cells. Both effects were restricted to only a fraction of cells in an all-or-none fashion. While the loss of IL-6 inducible STAT3 activation was permanent during the time course of the experiment, elevation of pSer-STAT3 level appeared to be a transient effect. We were able to detect a subpopulation of cells where both types of STAT3 phosphorylation were affected by chelidonine. Chelidonine reduced cell surface expression of gp130, but did not alter that of IL-6Rα or the level of total STAT3.

Taken together, our results demonstrated the involvement of STAT3 signaling in the mechanism of action of chelidonine in human uveal melanoma cells, which raises the applicability of this alkaloid in the combinational or multimodal therapies of uveal melanomas and possibly other solid tumors. In addition, we also demonstrated that techniques capable of providing cell-by-cell data might extend our understanding of signaling processes.

## 2. Results

### 2.1. Chelidonine Abrogates IL-6-Induced Activation of STAT3 in Uveal Melanoma Cells

To investigate the potential impact of chelidonine on the activation (i.e., tyrosine phosphorylation) of STAT3, OCM-1 and OCM-3 uveal melanoma cells were cultured for 24 h in the presence of chelidonine or DMSO alone (vehicle control) and then stimulated with IL-6. Efficiency of STAT3 activation was assessed by flow cytometry detecting the binding of anti-pTyr-STAT3 mAbs.

**Figure 1:**
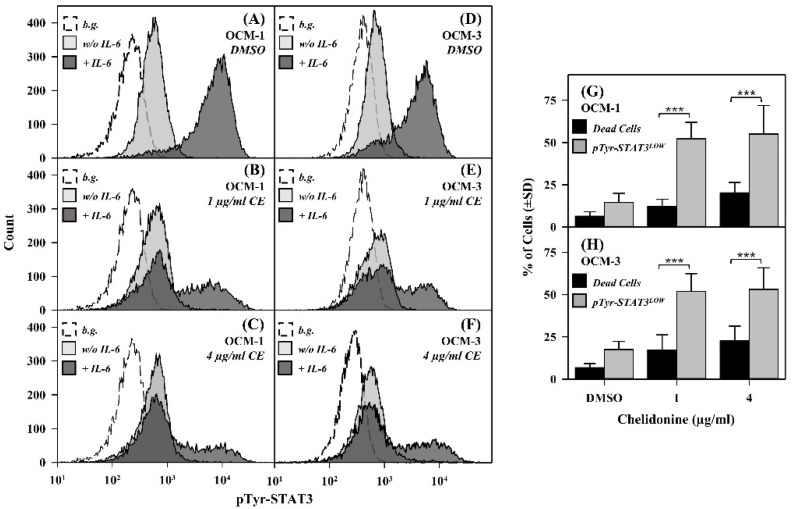
IL-6 induced tyrosine phosphorylation of STAT3 is abolished completely by chelidonine in a significant portion of OCM-1 and OCM-3 human uveal melanoma cells. (**A**–**F**) Representative flow cytometric intensity histograms demonstrate cell-by-cell distribution of pTyr-STAT3 levels in DMSO- and chelidonine-treated cells. Dark and light grey histograms correspond to IL-6-stimulated and unstimulated cells, respectively, whereas the empty histograms represent unlabeled cells (background). (**G**,**H**) Bar charts demonstrate the percentage of IL-6 nonresponsive cells and that of dead cells (grey and black bars, respectively) for DMSO- and chelidonine-treated OCM-1 and OCM-3 cell lines. The values for dead cells represent the fraction of apoptotic and necrotic cells combined. Percentages are expressed as the mean ± SD values of at least three independent experiments, *p*-value < 0.001 (***). The fraction of IL-6 nonresponsive cells increased significantly upon 1 or 4 µg/mL chelidonine treatment as compared to control (DMSO-treated) samples for both cell lines (*p* < 0.001, not indicated on the figure). Cells treated with chelidonine (1 or 4 µg/mL) or DMSO (vehicle control) for 24 h were incubated either in the presence of IL-6 (20 ng/mL) or alone for 30 min at 37 °C. Cells were then subjected to immunofluorescence staining using Alexa Fluor 647-conjugated mAbs specific for pTyr-STAT3, and analyzed by flow cytometry (n = 10,000 cells/sample). Bar charts: The percentage of IL-6 nonresponsive cells was determined by the evaluation of flow cytometric data detected for IL-6 stimulated cells as described in the Materials and Methods. The fraction of dead cells was determined based on Annexin-V and PI staining of DMSO- or chelidonine-treated cells without IL-6 stimulation. (b.g.: background, CE: chelidonine, pTyr-STAT3: STAT3 phosphorylated on the tyrosine 705 residue, w/o: without).

IL-6 evoked tyrosine phosphorylation of STAT3 in ~85% and ~80% of control (DMSO-treated) OCM-1 and OCM-3 cells, respectively (Figure 1). A fraction of cells remained nonresponsive in terms of STAT3 activation in both cell lines. Chelidonine exerted a bipartite effect; while it significantly increased the portion of cells with reduced responsiveness to IL-6 in terms of STAT3 activation, the rest of the cells retained their responsiveness. Cells still responsive to IL-6 after chelidonine treatment displayed IL-6-induced pTyr-STAT3 levels similar to those of cells not treated with chelidonine, as demonstrated by the flow cytometry intensity histograms shown in Figure 1. The basal level of pTyr-STAT3 (in cells not treated with IL-6) was not significantly affected by the alkaloid (Figure 1A–F). As it is shown in the Supplementary section (Figure S1), DMSO did not influence the activation pattern of STAT3; therefore, the effects observed upon chelidonine treatment could be entirely attributed to the alkaloid.

Chelidonine caused cell death in the doses applied in our experiments in both cell lines [20]. However, the fraction of dead cells was significantly lower than that of cells with reduced pTyr-STAT3 level, even for the higher concentration of the alkaloid (4 µg/mL); i.e., it can be excluded that abrogated responsiveness to IL-6 simply reflects the reduced cell viability (Figure 1). Total STAT3 levels in the vast majority of cells did not change upon chelidonine treatment (Figure S2); therefore, decreased expression of STAT3 can also be ruled out behind the observed effect.

### 2.2. Chelidonine Increases the Basal Level of Serine-Phosphorylated STAT3

**Figure 2:**
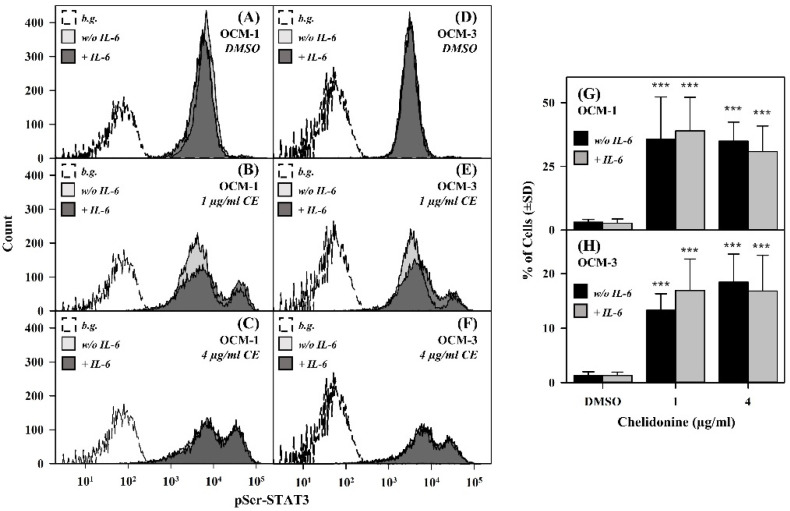
Serine phosphorylation of STAT3 is enhanced by chelidonine in a significant portion of OCM-1 and OCM-3 cells. (**A**–**F**) Representative flow cytometric intensity histograms demonstrate cell-by-cell distribution of pSer-STAT3 levels in DMSO- and chelidonine-treated cells. The thick black line and the filled grey histograms belong to IL-6-stimulated and unstimulated cells, respectively, whereas the dashed line histograms represent unlabeled cells (background). (**G**,**H**) Bar charts demonstrate the fraction of cells with increased levels of pSer-STAT3 for DMSO- and chelidonine-treated OCM-1 and OCM-3 cells. Grey and black bars represent IL-6-stimulated and unstimulated cells, respectively. Percentages are expressed as the mean ± SD values of at least three independent experiments, *p*-value < 0.001 (***). Cells treated with chelidonine (1 or 4 µg/mL) or DMSO (vehicle control) for 24 h were incubated either in the presence of IL-6 (20 ng/mL) or alone for 30 min at 37 °C. Cells were then stained with PE-conjugated mAbs specific for pS727-STAT3 and analyzed by flow cytometry (n = 10,000 cells/sample). (b.g.: background, CE: chelidonine, pSer-STAT3: STAT3 phosphorylated on the serine 727 residue, w/o: without).

As a next step, we checked whether chelidonine influences phosphorylation of STAT3 on the serine 727 residue, a potential regulator of the transcriptional activity of STAT3, by using flow cytometry. Contrary to pTyr-STAT3, the basal level of pSer-STAT3 rose dramatically upon chelidonine treatment in a significant fraction of the cells (Figure 1 and Figure 2).

This effect could be observed in both cell lines; however, the fraction of affected cells was somewhat lower for OCM-3 cells. In the rest of the cells, the level of pSer-STAT3 was similar to those of non-treated samples. Addition of IL-6 only slightly (if at all) altered the level of serine phosphorylated STAT3 as compared to the unstimulated counterparts (Figure 2).

### 2.3. The Fractions of Cells with Reduced IL-6 Responsiveness and Elevated pSer-STAT3 Level Follow Distinct Kinetics, with an Increasing Partial Overlap between the Affected Subpopulations

**Figure 3:**
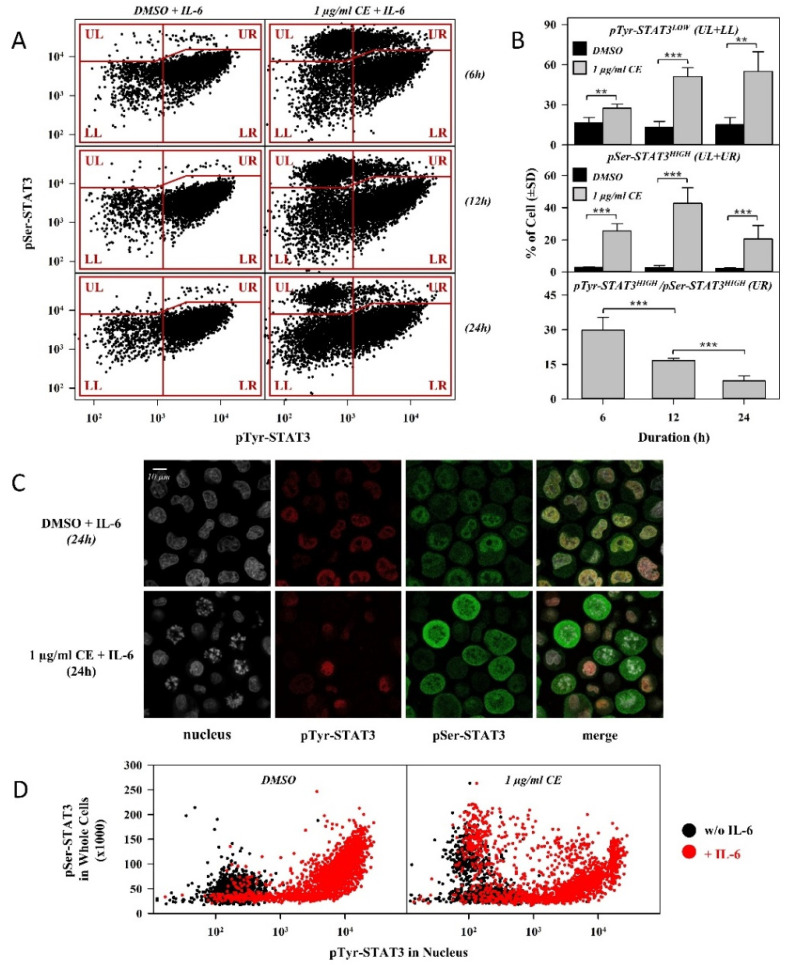
Subpopulations of OCM-1 cells with reduced IL-6 responsiveness and elevated pSer-STAT3 level overlap partially with each other. (**A**) Representative flow cytometry dot plots of pSer-STAT3 vs. pTyr-STAT3 levels in OCM-1 cells incubated for the indicated durations with chelidonine (right panels) or DMSO (left panels). n = 10,000 cells/sample were measured. Before analysis, cells were stimulated with IL-6 (20 ng/mL, 30 min) and labeled with Alexa Fluor 647- and PE-conjugated mAbs targeting pTyr-STAT3 and pSer-STAT3. (UL: upper left, UR: upper right, LL: lower left, LR: lower right) (**B**) Time dependence of the effects of chelidonine on the phosphorylation state of STAT3 in OCM-1 cells. The top and middle panels show the fraction of IL-6 unresponsive cells (in terms of STAT3 activation, pTyr-STAT3^LOW^) and that of cells with elevated pSer-STAT3 levels (pSer-STAT3^HIGH^), respectively, for DMSO- (black bars) and chelidonine-treated (grey bars) cells. The bottom panel depicts the fraction of cells with elevated pSer-STAT3 levels, induced by chelidonine treatment, which are still responsive to IL-6-induced tyrosine phosphorylation (pTyr-STAT3^HIGH^/ pSer-STAT3^HIGH^) (UL, UR, LL and LR refer to the respective fractions of cells in part A). The percentage of cells was determined with quantitative analysis of flow cytometry dot plots shown in part A. Percentages are expressed as mean ± SD values for at least three independent experiments, *p*-value < 0.001 (***) or < 0.01 (**). (**C**) Confocal microscopy images depict subcellular localization of pTyr- and pSer-STAT3 in DMSO- (top panels) and chelidonine-treated (bottom panels) cells. Images in the first column show nuclei stained with DAPI (grey), whereas images in the second and third columns represent subcellular distribution of pTyr-STAT3 (red) and pSer-STAT3 (green). Overlay images (last column) show co-localization of the labels. For microscopy experiments, cells were cultured in the presence of chelidonine or DMSO for 24 h and then processed as described in part A. (**D**) Representative dot plots demonstrate the correlated levels of nuclear pTyr-STAT3 and whole cell pSer-STAT3 in DMSO- (left panel) and chelidonine-treated cells (right panel) with and without IL-6 stimulation (red and black dots, respectively). Fluorescence intensities, representing average values of single nuclei or single cells, were derived from at least 40 images (number of analyzed cells > 1500), for which a representative series is shown in part B, after segmentation. (CE: chelidonine, pTyr- and pSer-STAT3: STAT3 phosphorylated on the tyrosine 705 and serine 727 residues, respectively).

Next, we checked the time dependency of the effects exerted by chelidonine on serine and tyrosine phosphorylation of STAT3, and the potential correlation between the two effects. The concomitant binding of mAbs, specific to the two types of phosphorylated STAT3, was followed by flow cytometry at three time points. The dot plots in Figure 3A show the correlated levels of pTyr- and pSer-STAT3 for a representative experiment in cells cultured with chelidonine (or DMSO alone) for 6, 12 and 24 h, and then stimulated with 20 ng/mL IL-6 for 30 min. Chelidonine was used at a concentration of 1 µg/mL to minimize cell death.

We have demonstrated that, similar to the 24-h treatment, chelidonine abrogated IL-6-induced tyrosine phosphorylation of STAT3 and increased the basal level of pSer-STAT3 in a significant portion of cells after the shorter incubations. Responsiveness to IL-6 decreased monotonously with time upon chelidonine treatment as shown by the increase of the fraction of cells with low tyrosine phosphorylation (pTyr-STAT3^LOW^) (Figure 3A UL and LL quadrants, Figure 3B top panel). On the other hand, the fraction of cells with elevated pSer-STAT3 levels (pSer-STAT3^HIGH^) exhibited biphasic behavior with the duration of chelidonine treatment; it increased up to ~40% after 12 h, followed by a backdrop to ~20% after 24 h (Figure 3B middle panel, Figure 3A UL and UR quadrants). The observed changes were statistically significant both for the 6-h vs. 12-h and for the 12-h vs. 24-h comparisons (*p* < 0.001, not indicated on Figure 3B). Based on our data it can also be concluded that chelidonine exerts an all-or-nothing effect on both types of phosphorylation, regardless of the duration of the treatment (Figure 3A).

After 24 h chelidonine treatment, the majority of pSer-STAT3^HIGH^ cells had low tyrosine phosphorylation, meaning that they were unresponsive to IL-6 stimulation (Figure 3A, lowermost panel), indicating a possible correlation between the effects of chelidonine. These data were corroborated by the microscopic inspection of cells (Figure 3C,D), showing an increase of the pTyr-STAT3^LOW^ fraction as well as a decreased tendency of nuclear translocation of pTyr-STAT3 in cells with elevated pSer-STAT3 levels. Further representative microscopic images, including images recorded for IL-6-nonstimulated cells, are provided in the Supplementary section (Figure S3). However, a significant fraction of cells with an elevated basal pSer-STAT3 level was still responsive to IL-6 stimulation in the case of shorter incubations (Figure 3A UR quadrant); this fraction decreased monotonously with time (Figure 3B, bottom panel). It should be noted that there is an IL-6-nonresponding (pTyr-STAT3^LOW^) subpopulation among those cells whose pSer-STAT3 levels were not increased by chelidonine (Figure 3A, LL quadrant).

### 2.4. Chelidonine Modifies the Expression of IL-6/gp130 Receptor Subunits

**Figure 4:**
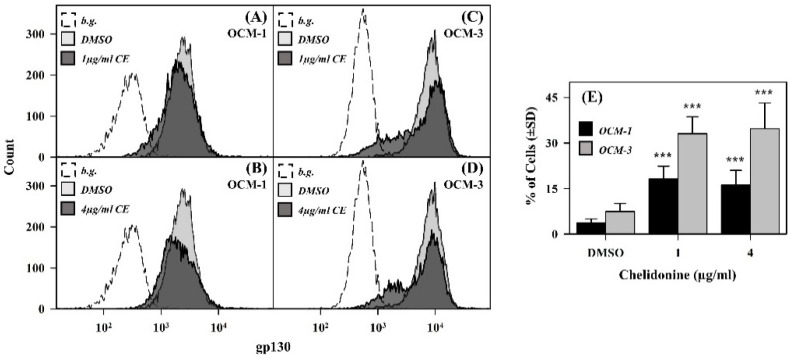
Chelidonine decreases the expression of gp130 in the cell membrane in OCM-1 and OCM-3 cell lines. (**A**–**D**) Representative flow cytometric histograms demonstrating the expression level of gp130 receptor subunits in DMSO- (light grey) and chelidonine-treated (dark grey) cells. The unlabeled DMSO-only-treated cells are depicted as empty dashed histograms. (**E**) Percentages of cells with reduced gp130 expression (OCM-1: black bars; OCM-3: grey bars). Percentages are expressed as mean ± SD values for at least three independent experiments, *p* < 0.001 (***). Cells treated with chelidonine (1 or 4 µg/mL) or DMSO for 24 h were stained with PE-conjugated mAb specific for gp130 and analyzed by flow cytometry (n = 10,000 cells/sample). (b.g.: background, CE: chelidonine).

We also analyzed the effect of chelidonine on the expression levels of gp130 and IL-6Rα, the two subunits of the functional IL-6 receptor, in both cell lines (Figure 4).

According to our flow cytometry results, the expression level of gp130 decreased in a significant portion of both cell types upon chelidonine treatment after 24 h (Figure 4). The percentage of affected cells was approximately twice as much in OCM-3 as in OCM-1 cells. At the same time, the alkaloid did not affect the expression of the IL-6Rα subunits significantly (Figure S4).

## 3. Discussion

Herb-derived natural products, interfering with STAT3 signaling in tumor cells, are promising therapeutic agents in cancer treatment [31]. Many of them are already in use, whereas others are undergoing clinical trials. Here we show that chelidonine, the major alkaloid component of *Chelidonium majus* L., exerts opposing effects on IL-6-induced activation and constitutive serine phosphorylation of STAT3 in human uveal melanoma cells. Chelidonine was found to reduce the viability of numerous cancer cell types, to bind weakly to tubulin, and to inhibit microtubule polymerization [20,21,22,23,24]. The microtubule stabilizer paclitaxel and MT-destabilizing vinorelbine were shown previously to inhibit both constitutive and cytokine-induced tyrosine phosphorylation of STAT3 with a concomitant increase in the extent of its constitutive serine phosphorylation in human breast, ovarian and prostate cancer cell lines [29,30]. Nocodazole, which is also a MT-destabilizing agent, changed the constitutive level of both types of STAT3 phosphorylation in HeLa and transfected HEK293 cells [14]. Our results on the effects of chelidonine are in good accordance with the above-mentioned findings. With the exception of constitutive tyrosine phosphorylation, which remained unaffected in the investigated uveal melanoma cells, chelidonine influenced both IL-6 induced activation and constitutive serine phosphorylation of STAT3 in a similar fashion.

Contrary to the cited data, which were derived from isolates of bulk cell suspensions, our flow cytometric approach made it possible to assess the effects of chelidonine on a cell-by-cell basis. This way we were able to demonstrate that the effects evoked by chelidonine are not homogenous throughout the entire cell population; instead, only a fraction of cells was affected even after the longest treatment applied, in an “all-or-none” fashion (i.e., the phosphorylation profile of STAT3 was either affected or remained unchanged). Chelidonine affected tyrosine and serine phosphorylation of STAT3 in distinct ways. The fraction of cells with abrogated capability for STAT3 tyrosine phosphorylation increased monotonously with time and persisted for the duration of our experiments, whereas the effect on serine phosphorylation was shorter-term; the percentage of pSer-STAT3^HIGH^ cells eventually declined after the initial rise.

The level of pSer-STAT3 coincided strongly with the activity of Cdk1 (a kinase capable of STAT3 serine phosphorylation) in serum-deprived HeLa cells released from the G0/G1 block, indicating that the increased level of pSer-STAT3 is a normal cell cycle event associated with the onset of mitosis [14]. It is plausible to assume that the transient effect exerted by chelidonine on the level of pSer-STAT3 in our experiments reflects the same cyclicality, i.e., chelidonine interferes with the machinery leading to activation of Cdk1. This hypothesis is supported by previous data demonstrating that the initial upregulation of cyclin B level, the activator for Cdk1, was followed by a decline upon chelidonine treatment in a gastric cancer cell line [21]. Furthermore, the level of pSer-STAT3 changed in a similar fashion in nocodazole-treated HeLa cells, where Cdk1 was active, whereas the total level of STAT3 did not change [14]. According to our microscopy data, chelidonine-induced serine phosphorylation of STAT3 presumably happens in the cytosol. Although Cdk1-cyclin B complexes translocate rapidly to the nucleus after being activated in the cytosol, a significant fraction of active complexes remains in the cytosol, which might account for the observed rise in the pSer-STAT3 level [32,33].

Integrating our own results with data available in the literature, it seems that (at least) two cellular pools of pSer-STAT3 exist, which probably rely on the action of different sets of kinases (and other regulators). A certain level of pSer-STAT3 (either constitutive or inducible) seems to be essential for the optimal canonical function of STAT3 in melanoma cells [34]. Consistent with these data, basal serine phosphorylation of STAT3 in the uveal melanoma cells investigated in our study did not prevent the activation and nuclear translocation of STAT3 under normal circumstances, i.e., without alkaloid treatment. The second pool of pSer-STAT3 is normally associated with the onset of mitosis, but can also be induced by different stress factors, e.g., UV-irradiation or MTAs [14,30,35]. According to our data, chelidonine affected the mechanisms contributing to this second pool of pSer-STAT3 only; those responsible for the original basal level of serine phosphorylation remained unaffected, as suggested by transiency of the effect. Chelidonine may switch on/accelerate the machinery required for serine phosphorylation of STAT3 (e.g., Cdk1), but probably does not alter negative regulators leading to dephosphorylation of STAT3 on the serine residue, which could account for the reversibility of the process. The percentage of cells in the G2/M phase of the cell cycle significantly exceeded the percentage of pSer-STAT3^HIGH^ cells for each time point confirming that elevated levels of pSer-STAT3 are present only in the initial phase of mitosis and are not required, at least directly, for the cell cycle arrest caused by chelidonine (Figure S5).

Combining flow cytometry and microscopy data, we demonstrated that elevated levels of pSer-STAT3 are accompanied with the abrogation of both IL-6-stimulated activation and subsequent nuclear translocation of STAT3 in the majority of affected cells after 24 h chelidonine treatment. These data suggest a potential correlation between the observed effects of chelidonine on the two types of STAT3 phosphorylation. A significant fraction of pSer-STAT3^HIGH^ cells could still respond to IL-6 stimulation with STAT3 activation after the shortest (6-h) treatment, indicating that the effects responsible for augmented serine phosphorylation precedes those leading to abrogated IL-6 responsiveness. Consequently, direct connection between the two effects can probably be excluded. The fraction of IL-6 responsive cells in the pSer-STAT3^HIGH^ subpopulation decreased monotonously with the duration of the treatment, making the postulated correlation more pronounced with time.

In light of the above, the question arises whether there is a causal relationship between the elevated serine and abrogated tyrosine phosphorylation of STAT3 elicited by chelidonine. UV-induced upregulation of pSer-STAT3 in parallel with the repression of STAT3 activation and function was demonstrated both in vitro and in vivo, but it was also hinted that the underlying mechanisms are probably independent of each other [35]. In accordance with this, inhibition of STAT3 activation by paclitaxel was observed even in the absence of serine phosphorylation in cells expressing STAT3 with a mutated serine residue [30]. These data do not support causality, not to mention direct connection, between elevated serine and suppressed tyrosine phosphorylation of STAT3 upon chelidonine treatment, therefore warranting further investigations to elucidate this question.

We could also detect the lack of IL-6-induced STAT3 activation in some cells with normal levels of pSer-STAT3, supporting that elevated serine phosphorylation is not a prerequisite for abrogated tyrosine phosphorylation of STAT3. This subpopulation may represent cells for which the effect of chelidonine on the level of pSer-STAT3 had already been reversed.

Reduced expression of gp130 observed in a significant portion of cells upon chelidonine treatment may provide an explanation for IL-6 unresponsiveness in terms of STAT3 activation. However, the portion of these cells is significantly lower than that of IL-6 unresponsive ones, especially in the case of the OCM-1 cell line. Furthermore, these cells still express gp130, which, together with the unchanged level of IL-6Rα, suggests that they still carry functional IL-6R complexes. Thus, reduced gp130 expression as a primary cause of IL-6 unresponsiveness could be excluded. However, it may change the sensitivity of cells to stimulation with IL-6 or other members of the IL-6 cytokine family; for example, IL-11 was observed in highly elevated concentrations in exosomes derived from primary uveal melanoma patients, with a further upregulation in the case of metastasis, indicating a role in uveal melanoma progression [36]. Activation of Erk2, controlling expression of gp130 [37], was recently shown to be inhibited by chelidonine, providing a possible mechanism for the reduced expression of gp130 [18,23].

Taken together, we have shown that chelidonine affects STAT3 signaling in the uveal melanoma cells studied in our experiments. Conventional biochemical methods previously used to study the serine and tyrosine phosphorylation of STAT3 did not allow studying of the correlation between the two parameters and lacked the potential to identify distinct subpopulations. Flow cytometry combined with microscopy allowed us to compare the two parameters on a cell-by-cell basis, examine their correlation, and distinguish subpopulations that respond differently to IL-6 and chelidonine treatment, which is important for understanding the antitumor mechanism of action.

Targeting STAT3 activity is a promising strategy both in combinational and multimodal approaches in cancer therapy [38,39]. Among others, inhibition of STAT3 was found to sensitize various types of tumor cells for radiation and chemotherapy [39]. Our results on chelidonine, along with its ability to overcome multidrug resistance, make this alkaloid a potential candidate for future combinational/multimodal therapeutic approaches [40].

## 4. Materials and Methods

### 4.1. Cell Cultures and Cytokine Treatment

OCM-1 and OCM-3 (ocular choroidal melanoma-1 and 3) human primary uveal melanoma cell lines (kindly provided by Dr. H.M.H. Hurks, Department of Ophthalmology, Leiden University Medical Center, Leiden, The Netherlands) were grown in RPMI 1640 medium (R6504, Sigma-Aldrich, St. Louis, MO, USA) supplemented with 5% fetal bovine serum (FBS, F9665, Sigma-Aldrich, St. Louis, MO, USA), 50 µg/mL penicillin (P4333, Sigma-Aldrich, St. Louis, MO, USA) and 2 mM L-glutamine (G3126, Sigma-Aldrich, St. Louis, MO, USA). Cells were cultured at 37 °C, in a humidified 5% CO_2_ atmosphere.

Cells were subcultured three times per week using the standard trypsinization method. In the case of IL-6 treatment, freshly harvested cells were washed in RPMI, suspended in fresh medium containing 20 ng/mL IL-6 (206-IL-010, R&D Systems, Minneapolis, MN, USA) at a cell concentration of 1 × 10^6^ cells/mL, and incubated for 30 min at 37 °C.

### 4.2. Treatment with Chelidonine

Chelidonine (54274, Sigma-Aldrich, St. Louis, MO, USA) was dissolved in dimethyl sulfoxide (DMSO) (D2650, Sigma-Aldrich, St. Louis, MO, USA). Before use, the stock solution was serially diluted with the solvent so that the final concentration of DMSO was the same in all related samples (5 µL/mL). Cells were seeded in 6-well-plates (~5 × 10^5^ cells/well in 2 mL medium). Cells grown to ~80–90% confluency (approx. 24 h after seeding) were treated with chelidonine (1 or 4 µg/mL) and cultured for the indicated duration afterwards. Control cells were treated with the same amount of DMSO and kept at the same experimental conditions. After alkaloid treatment, cells were harvested and prepared for further investigations as described later. Due to alkaloid treatment, a fraction of cells detached from the surface of the culture plate and floated in the medium. In order to avoid the loss of detached cells, the supernatant was collected before trypsinization. Cells retrieved from the supernatant by centrifugation were pooled with the trypsinized ones thereafter.

### 4.3. Immunofluorescence

Tyrosine and serine phosphorylation of STAT3 was followed by detecting the binding of Alexa Fluor 647 conjugated phospho-Tyr705-STAT3 specific (mouse IgG2a, 557815, BD Biosciences, Franklin Lakes, NJ, USA) and phycoerythrin (PE)-conjugated phospho-Ser727-STAT3 specific (mouse IgG1, 558557, BD Biosciences, Franklin Lakes, NJ, USA) mAbs, respectively. Relative expressions of total STAT3, IL-6Rα and gp130 were assessed using PE-conjugated anti-STAT3 (mouse IgG2B, IC1799P, R&D Systems, Minneapolis, MN, USA), AlexaFluor 647- or PE-conjugated anti-IL-6Rα (mouse IgG1, 352804, BD BioLegend, San Diego, CA, USA) and PE-conjugated anti-gp130 (mouse IgG2a, 362004, BD BioLegend, San Diego, CA, USA) mAbs, respectively. Nonspecific binding of monoclonal antibodies was checked using irrelevant antibodies with the same isotype. Since the differences between the fluorescence intensity histograms of the isotype controls and the unstained cells were negligible, the unstained samples were used as background in flow cytometry experiments.

#### Labeling Cells with Fluorescent Antibodies

For cell surface labeling, cells treated with chelidonine or DMSO and processed as described in Section 4.2 were washed twice in ice-cold phosphate buffered saline (PBS, pH 7.4). The cell pellet was suspended in PBS and incubated with saturating concentrations of fluorescent mAbs for 40 min on ice. After being washed, cells were fixed with 2% formaldehyde/PBS. Special care was taken to keep the cells at ice-cold temperature to avoid receptor internalization.

For intracellular labeling, cells were fixed in 2% formaldehyde/PBS (10 min at 37 °C) and then permeabilized in 90% methanol (30 min on ice). Cells were washed twice, suspended in the staining buffer (PBS + 2% fetal bovine serum + 0.1% sodium azide) and stained with the mAbs (10^6^ cells/50 µL for 45 min at room temperature). After staining, cells were washed twice in PBS and fixed with 2% formaldehyde in PBS.

### 4.4. Flow Cytometry

Flow cytometric analysis was performed on a FACSArray flow cytometer (Becton Dickinson Company, Franklin Lakes, NJ, USA) equipped with green (532 nm) and red (635 nm) diode lasers. Alexa Fluor 546, PI and PE were excited by the green laser, whereas for the excitation of Alexa Fluor 647, the red laser was used. Fluorescence emissions of Alexa Fluor 546, PI and PE were detected through a 564–576-nm, while that of Alexa Fluor 647 through a 553–679-nm band-pass filter. Samples prepared in triplicates were measured within 24 h after preparation and analyzed using the FCS Express (De Novo Software, Pasadena, CA, USA) and FlowJo (BD Biosciences, Franklin Lakes, NJ, USA) software tools.

During the evaluation of flow cytometric data for doubly labeled cells, a reference gate was determined in the appropriate fluorescence intensity dot-plot that contained the majority of cells of the untreated sample. In a treated sample, cells outside the reference gate were taken as chelidonine- and/or IL-6 responsive cells. In the case of samples labeled by a single fluorophore, the fluorescence intensity–forward scatter dot-plot was used for analysis.

### 4.5. Detection of Cell Death

Cells treated with chelidonine or DMSO alone were processed as described in Section 4.2, then washed in PBS. The pellet was suspended in the Annexin V binding buffer (10 mM HEPES/NaOH, 0.14 M NaCl, 2.5 mM CaCl_2_, pH 7.5) at a concentration of ~10^6^ cells/mL. A volume of 100 µL of the cell suspension per assay was stained with 5 µL of Annexin V-Alexa Fluor 647 (640912, BD Biolegend, San Diego, CA, USA) and 0.5 µg/mL PI (P4170, Sigma-Aldrich, St. Louis, MO, USA) for 15 min at room temperature and then analyzed by flow cytometry (see Section 4.4). Live, apoptotic and necrotic cells were distinguished on the basis of their Annexin V reactivity and PI exclusion. Live, non-apoptotic cells were not stained with any of the reagents. Apoptotic cells exhibited intense Annexin V-Alexa Fluor 647 and low PI fluorescence, while the necrotic cells exhibited strong fluorescence intensity in both channels.

### 4.6. Confocal Laser Scanning Microscopy

For microscopy measurements, 4′,6-diamidino-2-phenylindole (DAPI, 0.5 µg/mL, 62248, Thermo Fisher Scientific, Waltham, MA, USA) was added to the staining mixture in order to visualize cell nuclei parallel with phosphorylated STAT3. Samples processed as described in 4.3.1 were mounted on poly-L-lysine-coated cover slips with Mowiol 4–88 (81381, Sigma-Aldrich, St. Louis, MO, USA) dissolved in glycerol to reduce unwanted photobleaching.

Subcellular localization of pTyr- or pSer-STAT3 was assessed on a Zeiss LSM 880 confocal microscope (Zeiss, Oberkochen, Germany). For the excitation of DAPI (cell nucleus), a 405-nm diode laser was used—for Alexa Fluor 488, the 488-nm line of an Argon ion laser; for PE, a 543-nm He-Ne laser; and for Alexa Fluor 647, a 633-nm He-Ne laser. Fluorescence emissions were detected through 410-474-nm, 499-533-nm, 562-615-nm and 651-755-nm band-pass filters, respectively. Images of approx. 1 μm thick optical sections, each containing 2412 × 2412-pixels (pixel size ~88 nm), were obtained with a 60× UPLSAPO oil immersion objective (NA 1.35) from the middle plane of the cells. Images were taken in sequential mode to minimize cross-talk between the channels.

#### Quantitative Analysis of Nuclear Translocation: Image Segmentation and Evaluation

To determine the fluorescence intensities characterizing pTyr- and pSer-STAT3 content in the whole cell or in the nucleus, nuclear and whole-cell masks were generated by using the Trainable Weka Segmentation plugin in FIJI [41]. Briefly, the boundaries of nuclei were manually circumscribed in a couple of DAPI-stained images, whereas the boundaries of cells were manually identified in a couple of samples stained for pSer-STAT3 that labeled most of the cytoplasm. Holes were filled generating whole-cell and nuclear masks for the training datasets in which binary 1s and 0s correspond to the foreground (cell or nucleus) and to the background, respectively. These binary masks were fed into the WEKA machine-learning tool for training a classifier. These classifiers were used for batch processing all images, generating whole-cell and nuclear masks. The rest of the analysis was performed in Matlab (Mathworks, Natick, MA, USA). Mean intensities were evaluated in the whole-cell and nuclear masks of individual cells. Mean intensities were background-corrected by subtracting the mean intensity of a region of interest manually drawn in each image in a cell-free area.

### 4.7. Statistical Analysis

Results are expressed as the means ± SD values. The mean values were compared using *t* tests for independent samples (homoscedastic or heteroscedastic versions depending on the results of equal variance test). Changes were considered to be significant for *p*-values smaller than 5%.

## Data Availability

The data presented in this study are available on request from the corresponding author.

## Supplementary Materials

The following are available online at https://www.mdpi.com/article/10.3390/ijms222312974/s1.

## Abbreviations

IL-6: interleukin-6; STAT3: signal transducer and activator of transcription; gp130: glycoprotein 130; IL-6R: interleukin-6 receptor; Jak: Janus kinase; MTAs: microtubule-targeting agents; pSer-STAT3: serine-phosphorylated STAT3; pTyr-STAT3: tyrosine-phosphorylated STAT3.
