# Supplementary material for: Opposing Effects of Chelidonine on Tyrosine and Serine Phosphorylation of STAT3 in Human Uveal Melanoma Cells
Source: Int J Mol Sci. 2021 Nov 30;22(23):12974. doi: 10.3390/ijms222312974 (PMC8658041; doi:10.3390/ijms222312974)
Supplement: Supplementary file 1 [file ijms-22-12974-s001.zip › ijms-1429875-supplementary.pdf]

Opposing effects of chelidonine on tyrosine and serine phosphorylation of STAT3 in human uveal melanoma cells

István Csomós, Péter Nagy, Csenge Filep, István Rebenku, Enikő Nizsalóczki, Tamás Kovács, György Vámosi, LászlóMátyus, Andrea Bodnár

SUPPLEMENTARY FIGURES

SUPPLEMENTARY FIGURE S1.

DMSO did not significantly influence IL-6 induced activation of STAT3 in OCM-1 and OCM-3 human uveal melanoma cells.

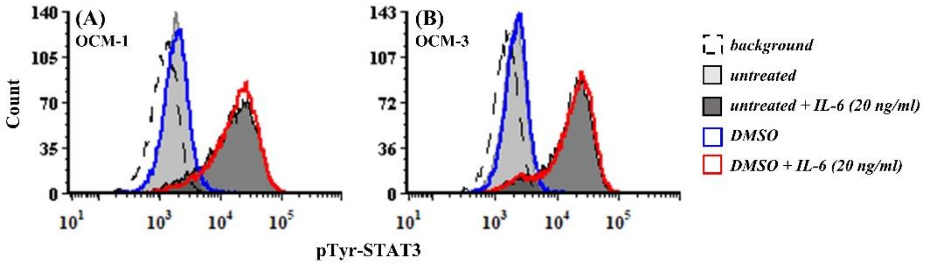

(A-B) Representative flow cytometric intensity histograms demonstrating cell-by-cell distribution of pTyr-STAT3 in IL-6-stimulated OCM-1 and OCM-3 cells cultured in the absence or presence of DMSO (dark grey and red histograms, respectively). Light grey and blue histograms belong to nonstimulated cells cultured without or with DMSO, respectively. The empty histograms represent unlabeled cells (background).

Cells were cultured with or without DMSO for 24 hours, then were incubated either in the presence of IL-6 (20ng/mL) or alone for 30 minutes at 37 °C. The same amount of DMSO (5 µl/mL) was applied as in the experiments with chelidonine. Cells were then subjected to immunofluorescence staining using Alexa Fluor 647-conjugated mAbs specific for pY705-STAT3, and analyzed by flow cytometry (n=10000 cells/sample). The same intracellular labeling protocol was applied as described in the Materials and Methods section of the manuscript. Alexa Fluor 647 fluorescence was excited by the red (635 nm) laser of a FacsArray flow cytometer and detected through a 553-679-nm band-pass filter. Samples were measured within 24 hours after preparation and analyzed using FCS Express.

SUPPLEMENTARY FIGURE S2.

Total STAT3 level is not altered significantly by chelidonine in OCM-1 and OCM-3 human uveal melanoma cells.

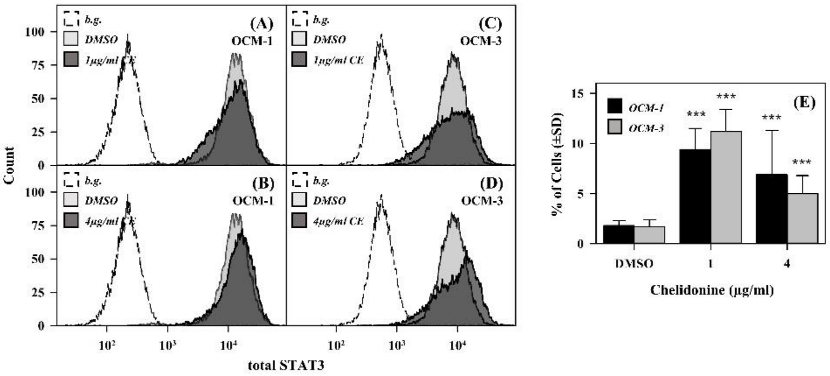

(A-D) Representative flow cytometric intensity histograms demonstrating the expression level of total STAT3 in DMSO- (light grey) and chelidonine-treated (dark grey) cells. The unlabeled DMSO-only-treated cells are depicted as empty dashed histograms. (E) Percentages of cells with reduced total STAT3 expression (OCM-1: black bars; OCM-3: grey bars). Percentages are expressed as mean ± SD values for at least three independent experiments, *p*-value < 0.001 (***). (b.g.: background, CE: chelidonine)

Cells treated with chelidonine (1 or 4μg/mL) or DMSO for 24 hours were stained with PE-conjugated mAb specific for STAT3 and analyzed by flow cytometry (n=10000 cells/sample). The same intracellular labeling protocol was applied as described in the Materials and Methods section of the manuscript. PE fluorescence was excited by the green (532 nm) laser of a FacsArray flow cytometer and detected through a 564-576-nm band-pass filter. Samples prepared in triplicates were measured within 24 hours after preparation and analyzed using FCS Express.

SUPPLEMENTARY FIGURE S3

Representative confocal microscopy images depicting subcellular localization of pTyr-STAT3 and pSer-STAT3 in DMSO- and chelidonine-treated OCM-1 cells.

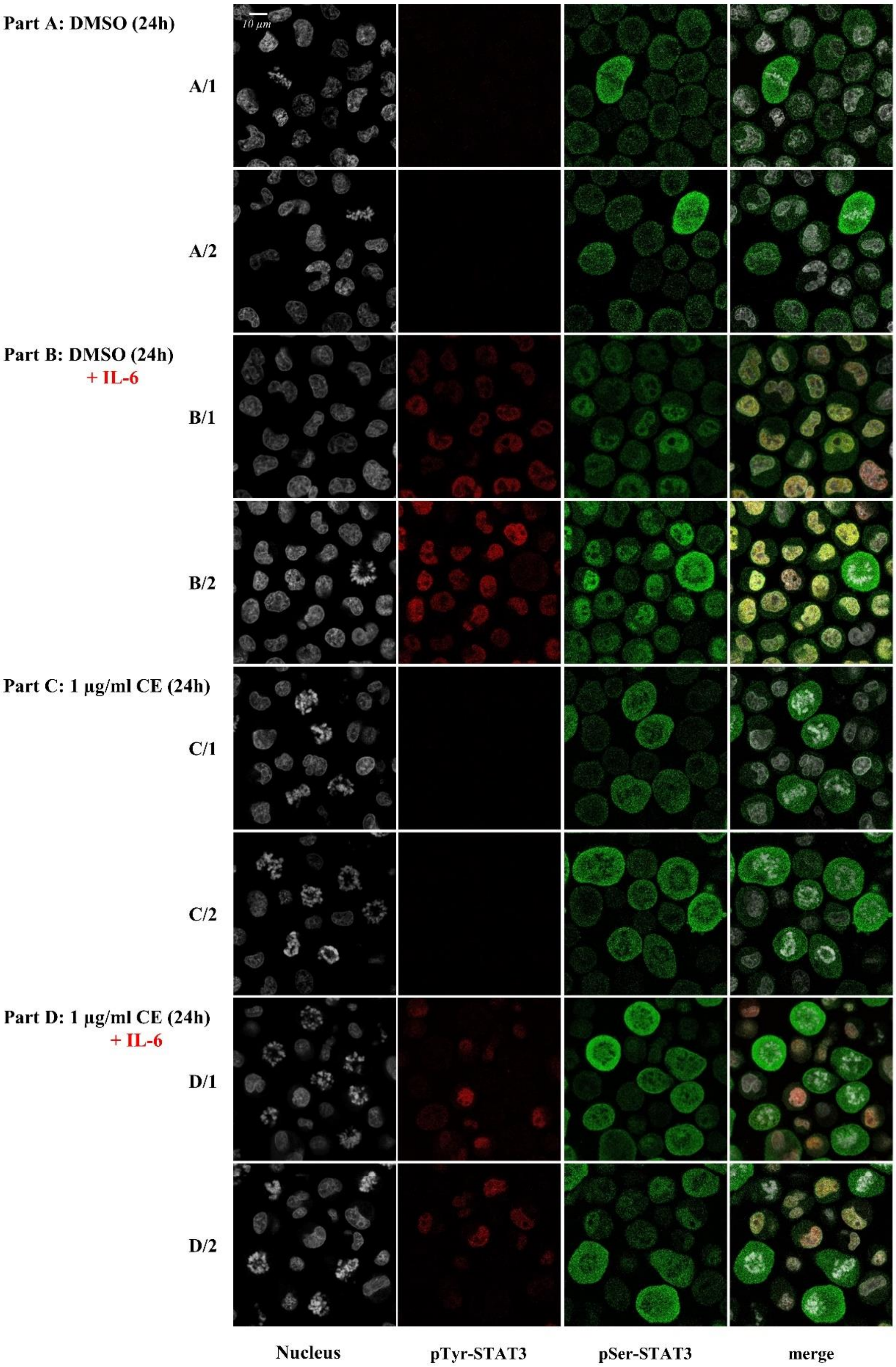

Part A and B correspond to nonstimulated (A/1 and A/2) and IL-6-stimulated (B/1 and B/2) control (DMSO-treated) OCM-1 cells, respectively. Part C and D depict nonstimulated (C/1 and C/2) and IL-6-stimulated (D/1 and D/2) chelidonine-treated cells, respectively. Panels B/1 and D/1 are the same images shown in Figure 3C of the manuscript. Images in the first column show nuclei stained with DAPI (grey), whereas images in the second and third columns represent the subcellular distribution of pTyr-STAT3 (red) and pSer-STAT3 (green), respectively. Overlay images (last column) show co-localization of the labels. Cells were cultured in the presence of chelidonine (1μg/mL) or DMSO (vehicle control) for 24 hours. Before confocal microscopic measurement, cells were incubated either in the presence of IL-6 (20ng/mL) or alone for 30 minutes at 37ºC and labeled with Alexa Fluor 647 and PE-conjugated mAbs targeting pTyr-STAT3 and pSer-STAT3, respectively. The same intracellular labeling protocol was applied as described in the Materials and Methods section of the manuscript. Subcellular localization of pTyr- or pSer-STAT3 was assessed on a Zeiss LSM 880 confocal microscope. For the excitation of DAPI (cell nucleus) a 405-nm diode laser; for Alexa Fluor 488 the 488-nm line of an Argon ion laser; for PE a 543-nm He-Ne laser and for Alexa Fluor 647 a 633-nm He-Ne laser was used. Fluorescence emissions were detected through 410-474-nm, 499-533-nm, 562-615-nm and 651-755-nm band-pass filters, respectively. (CE: chelidonine, pTyr- and pSer-STAT3: STAT3 phosphorylated on the tyrosine 705 and serine 727 residues, respectively)

SUPPLEMENTARY FIGURE S4.

Cell surface expression of IL-6Rα is not altered significantly by chelidonine in OCM-1 and OCM-3 human uveal melanoma cells.

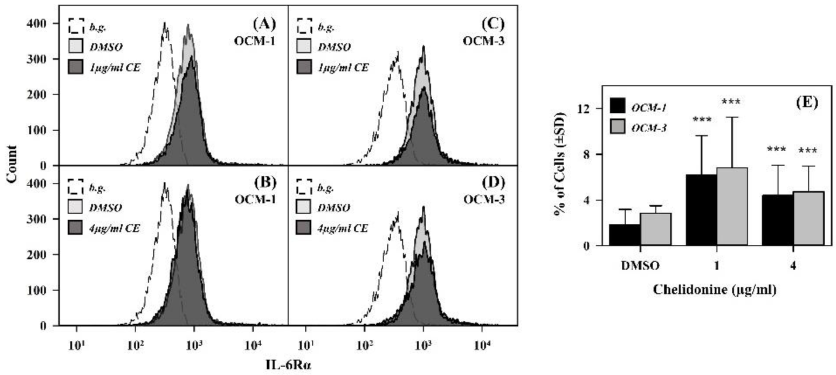

(A-D) Representative flow cytometric histograms demonstrating the expression level of IL-6Rα in DMSO- (light grey) and chelidonine-treated (dark grey) cells. The unlabeled DMSO-only-treated cells are depicted as empty dashed histograms. (E) Percentages of cells with reduced IL-6Rα expression (OCM-1: black bars; OCM-3: grey bars). Percentages are expressed as mean ± SD values for at least three independent experiments, *p*-value < 0.001 (***).

Cells treated with chelidonine (1 or 4 μg/mL) or DMSO for 24 hours were stained with PE-conjugated mAb specific for IL-6Rα and analyzed by flow cytometry (n=10000 cells/sample). The same cell surface labeling protocol was applied as described in the Materials and Methods section of the manuscript. PE fluorescence was excited by the green (532 nm) laser of a FacsArray flow cytometer and detected through a 564-576-nm band-pass filter. Samples prepared in triplicates were measured within 24 hours after preparation and analyzed using FCS Express. (b.g.: background, CE: chelidonine)

SUPPLEMENTARY FIGURE S5.

Chelidonine arrests uveal melanoma cells in the G2/M phase of the cell cycle.

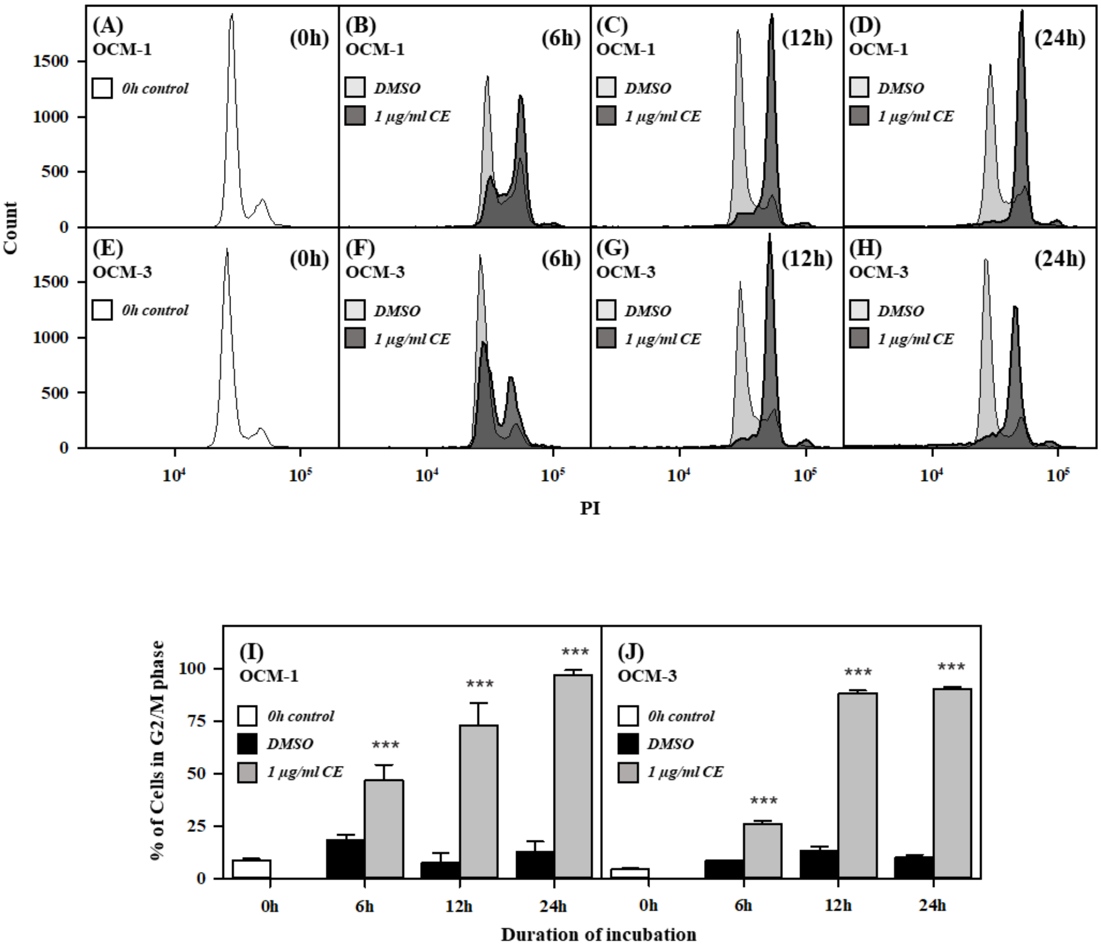

(A-H) Representative flow cytometric histograms demonstrate the DNA content/distribution in OCM-1 (A-D) and OCM-3 (E-H) cells. The untreated, freshly harvested cells are depicted as empty histograms (A and E). Light and dark grey histograms belong to DMSO-and chelidonine-treated cells, respectively, at the indicated times in hours. (I and J) The bar charts show the percentages of OCM-1 and OCM-3 cells in the G2/M phase (black bars: DMSO-treated cells; grey bars: chelidonine-treated cells). The empty bars show the fraction of G2/M phase cells for freshly harvested, untreated cells. Percentages are expressed as mean ± SD values for at least three independent experiments, p-value < 0.001 (***).

Cells treated with chelidonine (1 µg/mL) or DMSO for 6, 12 and 24 hours were permeabilized with PI-containing Triton-X hypotonic solution overnight and then their DNA content was analyzed by flow cytometry (n=10000 cells/sample). Cell cycle analysis was performed on a FACSArray flow cytometer (Becton Dickinson). PI fluorescence was excited by the green laser (532 nm) of the cytometer and detected through a 564-576-nm band-pass filter. Samples prepared in triplicates were measured within 24 hours after preparation and analyzed using the

built-in cell cycle analysis module of FCS express software. (CE: chelidonine, PI: propidium iodide)
